# Assessing human exposure to spotted fever and typhus group rickettsiae in Ontario, Canada (2013–2018): a retrospective, cross-sectional study

**DOI:** 10.1186/s12879-020-05244-8

**Published:** 2020-07-18

**Authors:** Mark P. Nelder, Curtis B. Russell, Steven Johnson, Ye Li, Kirby Cronin, Bryna Warshawsky, Nicholas Brandon, Samir N. Patel

**Affiliations:** 1 Enteric, Zoonotic and Vector-Borne Diseases; Communicable Diseases, Emergency Preparedness and Response, Public Health Ontario, Toronto, ON Canada; 2 Informatics, Knowledge Services, Public Health Ontario, Toronto, ON Canada; 3 Public Health Ontario Laboratory, Public Health Ontario, Toronto, ON Canada; 4 National Microbiology Laboratory, Public Health Agency of Canada, Winnipeg, MB Canada; 5 Department of Epidemiology and Biostatistics, Western University, London, ON Canada; 6 Dalla Lana School of Public Health, University of Toronto, Toronto, ON Canada; 7 Department of Laboratory Medicine and Pathobiology, University of Toronto, Toronto, ON Canada

**Keywords:** Epidemiology, Flea-borne, Public health, *Rickettsia*, Serology, Surveillance, Tick-borne, Vector

## Abstract

**Background:**

Assessing the burden of rickettsial infections in Ontario, Canada, is challenging since rickettsial infections are not reportable to public health. In the absence of reportable disease data, we assessed the burden of rickettsial infections by examining patient serological data and clinical information.

**Methods:**

Our retrospective, cross-sectional study included patients who had *Rickettsia* serological testing ordered by their physician, in Ontario, from 2013 to 2018. We tested sera from 2755 non-travel patients for antibodies against spotted fever group rickettsiae (SFGR) and typhus group rickettsiae (TGR) using an indirect immunofluorescence assay (IFA) (positive IgG titers ≥1:64). We classified cases using a sensitive surveillance case definition: confirmed (4-fold increase in IgG titers between acute and convalescent sera with clinical evidence of infection), possible (single positive sera with clinical evidence) and previous rickettsial infection (single positive sera without clinical evidence). We classified cases seropositive for both SFGR and TGR as unspecified *Rickettsia* infections (URIs).

**Results:**

Less than 5% of all patients had paired acute and convalescent sera tested, and of these, we found a single, laboratory-confirmed SFGR case, with a 4-fold increase in IgG titers and evidence of fever, maculopapular rash and headache. There were 45 possible (19 SFGR, 7 TGR, 19 URI) and 580 previous rickettsial infection (183 SFGR, 89 TGR, 308 URI) cases. The rate of positive tests for SFGR, TGR and URI combined (all case classifications) were 4.4 per 100,000 population. For confirmed and possible cases, the most common signs and symptoms were fever, headache, gastrointestinal complaints and maculopapular rash. The odds of having seropositive patients increased annually by 30% (odds ratio = 1.3, 95% confidence interval: 1.23–1.39).

**Conclusions:**

The rates of rickettsial infections in Ontario are difficult to determine. Based on confirmed and possible cases, rates are low, but inclusion of previous rickettsial infection cases would indicate higher rates. We highlight the need for education regarding the importance of testing acute and convalescent sera and consistent completion of the laboratory requisition in confirming rickettsial disease. We suggest further research in Ontario to investigate rickettsial agents in potential vectors and clinical studies employing PCR testing of clinical samples.

## Background

*Rickettsia* are Gram-negative, obligate, intracellular bacteria (Rickettsiales: Rickettsiaceae) organized into three groups based on shared phylogenetics, pathology, vectors and arthropod hosts: 1) spotted fever group rickettsiae (SFGR), 2) typhus group rickettsiae (TGR) and 3) ancestral group rickettsiae [1]. Ancestral group rickettsiae, unlike SFGR and TGR, are not associated with human disease. Transmission of rickettsiae to humans is usually through an arthropod bite; however, transmission can occur through inhalation of aerosolized bacteria (e.g., *Rickettsia prowazekii*) or through bacteria-laden arthropod feces entering the body at bite locations, conjunctiva or mucosa (e.g., *Rickettsia typhi*). SFGR and TGR infect the endothelial cells of blood vessels, causing vascular permeability and rash, followed by spread to other organs [2].

*Rickettsia rickettsii* causes Rocky Mountain spotted fever (RMSF) and is transmitted by several tick species, including American dog ticks (*Dermacentor variabilis*) and Rocky Mountain wood ticks (*Dermacentor andersoni*). RMSF is the most severe of the pathogenic SFGR and the case fatality rate is approximately 5–10%, if not properly treated [3]. The incubation period for SFGR is 2–28 days (≈ 7 days for *R*. *rickettsii*). SFGR infection causes a variety of signs and symptoms, including fever, chills, headache, malaise, myalgia, gastrointestinal complaints, mild leukopenia, thrombocytopenia and elevated hepatic transaminases [3–5]. Depending on the specific pathogen, patients may develop a maculopapular rash (e.g., *R*. *rickettsii*), vesiculopapular or pustular rash (*Rickettsia parkeri*), papulovesicular rash (*Rickettsia akari*) and/or an eschar at the inoculation site (*Rickettsia felis*, *R*. *akari*, *R. parkeri*) [6]. If untreated, RMSF can lead to cardiac, neurological and respiratory complications, with development of gangrene, sepsis or renal failure.

*Rickettsia prowazekii* causes epidemic typhus, a TGR transmitted by human body lice (*Pediculus humanus corporis*) and, in North America, maintained in a sylvatic cycle by southern flying squirrels (*Glaucomys volans*) and their lice (*Neohaematopinus scuiropteri*). *Rickettsia typhi*, another TGR, causes murine typhus and is transmitted by rodent and cat fleas (*Xenopsylla cheopis* and *Ctenocephalides felis*, respectively). Most TGR outbreaks are associated with 1) crowded conditions where body lice proliferate, usually in areas experiencing natural disasters, wars or famines or 2) urban and suburban populations encountering rodent or cat fleas [7–9]. Murine typhus has a case fatality rate of 4% when not treated, whereas untreated epidemic typhus has a case fatality rate of up to 60% [8, 10]. The incubation period for TGR is 7–14 days, with signs and symptoms including fever, maculopapular rash, eschar, chills, myalgia, arthralgia, headache, gastrointestinal complaints, cough and lymphadenopathy [8, 11]. Complications of untreated TGR are similar to those of RMSF noted above.

Rickettsial infections are not reportable to public health officials in Ontario; however, the vectors are present in the province (Table 1) [22]. Climate change may increase the risk of rickettsial diseases in the province through the range expansion of tick vectors, increased abundance of ticks and a lengthened active season of ticks and their hosts [23–25]. As Ontario has experienced with the expanding distribution of blacklegged ticks (*Ixodes scapularis*) and their pathogens, there is a potential for rickettsial disease emergence with changing distributions of *D. variabilis* and *Amblyomma americanum* [24, 26–28]. SFGR infections in the USA have increased from 1713 reported cases in 2004 to 4269 in 2016, potentially increasing the risk in Canada [29]. Currently, the only published reports of rickettsial infections in Ontario are travel related, including *Rickettsia africae* (African tick-bite fever) in travellers returning from Africa [30]. We assessed the potential burden of rickettsial infections in Ontario by using laboratory serological results from specimens submitted for rickettsiae testing from 2013 to 2018 and used the accompanying laboratory requisition data to obtain clinical information.

**Table 1:** Rickettsiae reported from Ontario, or associated with vectors, or inveterate hosts present in Ontario

Rickettsiae^a^	Disease	Potential Ontario vectors or invertebrate hosts
Spotted fever group		
*Rickettsia akari*	Rickettsialpox	**Mites:***Liponyssoides sanguineus*
*Rickettsia felis*	Flea-borne spotted fever	**Fleas: ***Ctenocephalides felis*
*Rickettsia montanensis*	Unknown pathogenicity^b^	**Ticks:***Amblyomma americanum*, *Dermacentor variabilis*
*Rickettsia peacockii*	Endosymbiont, non-pathogenic	**Ticks:***D. variabilis*
*Rickettsia rickettsii*	Rocky Mountain spotted fever	**Ticks:***A. americanum*, *D*. *variabilis*
Typhus group		
*Rickettsia typhi*	Murine typhus	**Fleas: ***C. felis*
Ancestral group		
*Rickettsia canadensis*	Endosymbiont, non-pathogenic	**Ticks: ***Haemaphysalis leporispalustris*

^a^ Selected references [5, 12–20]. Not included are rickettsiae detected in adventitious ticks collected in Ontario (*Rickettsia parkeri* in *Amblyomma maculatum*) [16]

^b^While human infections have been associated with this *Rickettsia* species, investigators did not confirm the etiological agent involved [21]

## Methods

### Study location

Ontario is located in the Great Lakes region of North America and is the most populous province in Canada (≈ 14.3 million) [31]. Most of Ontario’s population is concentrated in the southern portion of the province (south of 45°N), an area dominated by a moderate, humid, continental climate with a mixture of agricultural, suburban and urban landscapes.

Public health units (PHU) administer public health services in Ontario. During the study, there were 36 PHUs; however, we performed analyses on a dataset using the updated classification of 35 PHUs. ALG, Algoma District; BRN, Brant County; CHK, Chatham-Kent; DUR, Durham Regional; EOH, Eastern Ontario; GBO, Grey Bruce; HAL, Halton Regional; HAM, City of Hamilton; HDN, Haldimand-Norfolk; HKP, Haliburton-Kawartha-Pine Ridge District; HPE, Hastings and Prince Edward Counties; HUR, Huron County; KFL, Kingston-Frontenac and Lennox & Addington; LAM, Lambton; LGL, Leeds-Grenville and Lanark District; MSL, Middlesex-London; NIA, Niagara Regional; NPS, North Bay Parry Sound District; NWR, Northwestern; OTT, City of Ottawa; OXE, Oxford Elgin-St. Thomas; PDH, Perth District; PEL, Peel Regional; PQP, Porcupine; PTC, Peterborough County-City; REN, Renfrew County and District; SMD, Simcoe Muskoka District; SUD, Sudbury and District; THB, Thunder Bay District; TOR, City of Toronto; TSK, Timiskaming; WAT, Waterloo; WDG, Wellington-Dufferin-Guelph; WEC, Windsor-Essex County; YRK, York Regional.

### Sample population and *Rickettsia* serology

The sample population for this study included patients with *Rickettsia* serological testing ordered by their physician from January 1, 2013 through December 31, 2018 and submitted to the Public Health Ontario (PHO) laboratory. When physicians sent patient sera for serological testing, we requested follow-up convalescent sera collected 2–3 weeks later. In 155 patients, for which symptom onset date was available, the median time from symptom onset to collection of acute sera was 4 days (interquartile range: 1–10 days).

We performed *Rickettsia* serology using an indirect immunofluorescence assay (IFA) for SFGR and TGR antibodies. We screened for antibodies at a dilution of 1:64 using the Focus Diagnostics *Rickettsia* IFA IgG and IgM kits (Cypress, CA, USA). Samples that were reactive at a dilution of 1:64 were serially titrated 2-fold to determine the end-point titer (up to 1:256). Antibody reactivity to the *R*. *rickettsii* antigen is considered positive for SFGR (includes *R*. *rickettsii, R*. *africae, Rickettsia conorii, R. parkeri, R*. *akari* and *R*. *felis*), whereas antibody reactivity to the *R*. *typhi* antigen is considered positive for TGR (i.e., *R*. *prowazekii* and *R*. *typhi*). According to the IFA kit manufacturer, IFA IgG titers < 1:64 indicate no serologic evidence of infection, titers 1:64 to 1:128 provide serologic evidence of prior or recent infection and titers ≥1:256 provide evidence of current or recent infection. Data elements from the testing requisition included patient symptom onset date, date sample taken (which is used to calculate the estimated onset date when the patient symptom onset date was not provided), age, sex, PHU of residence, recent travel and signs and symptoms.

### Surveillance case classification

Confirmed cases demonstrated a 4-fold increase in IgG titers between acute and convalescent sera (2–3 weeks apart), with evidence of fever plus any other sign or symptom associated with rickettsial disease. Since clinical information is not fully captured on laboratory requisitions, we included a broad array of symptoms in conjunction with fever as clinical evidence of infection. A possible case demonstrated a single elevated IgG titer (≥ 1:64), with evidence of fever plus any other sign or symptom. A previous rickettsial infection case demonstrated a single elevated IgG titer (≥ 1:64), without evidence of the combination of fever plus any other sign or symptom. While single IgG titers ≥1:64 likely overestimate rickettsial infection, for this study we felt sensitivity was more important in our surveillance case definitions than specificity [32]. We did not assess IgM serological results for laboratory diagnoses, since, unlike for other infectious diseases, IgM is not useful in identifying current or recent rickettsial infections [3, 33, 34]. We classified cases seropositive for both SFGR and TGR as unspecified *Rickettsia* infections (URI), since the potential cross-reactivity of the test makes it difficult to determine which group is the cause of the infection.

### Statistical analyses and mapping

We used population data and estimates from Statistics Canada, via IntelliHEALTH Ontario, to calculate seropositive rates per 100,000 population (extracted October 19, 2017; 2018 data used a denominator). We used Excel v15.0 for statistical tests (ANOVA, chi-square, t-test) and considered a *p*-value < 0.05 statistically significant. We performed logistic regression using R v3.6.2 to study relationships between outcome and year, seasonality and PHU. Odds ratio was used to describe the strength of the association between year and outcome (being a case or not); i.e., as time progresses by year, and likelihood of outcome increases. The Wald test was used to exam the significance of year, and the likelihood ratio test was used to exam the significance of PHU and seasonality. We created maps using Esri ArcGIS v10.3, using manual classification methods to categorize PHU case rates.

## Results

### Case classification - serology and clinical presentation

**Fig. 1:**
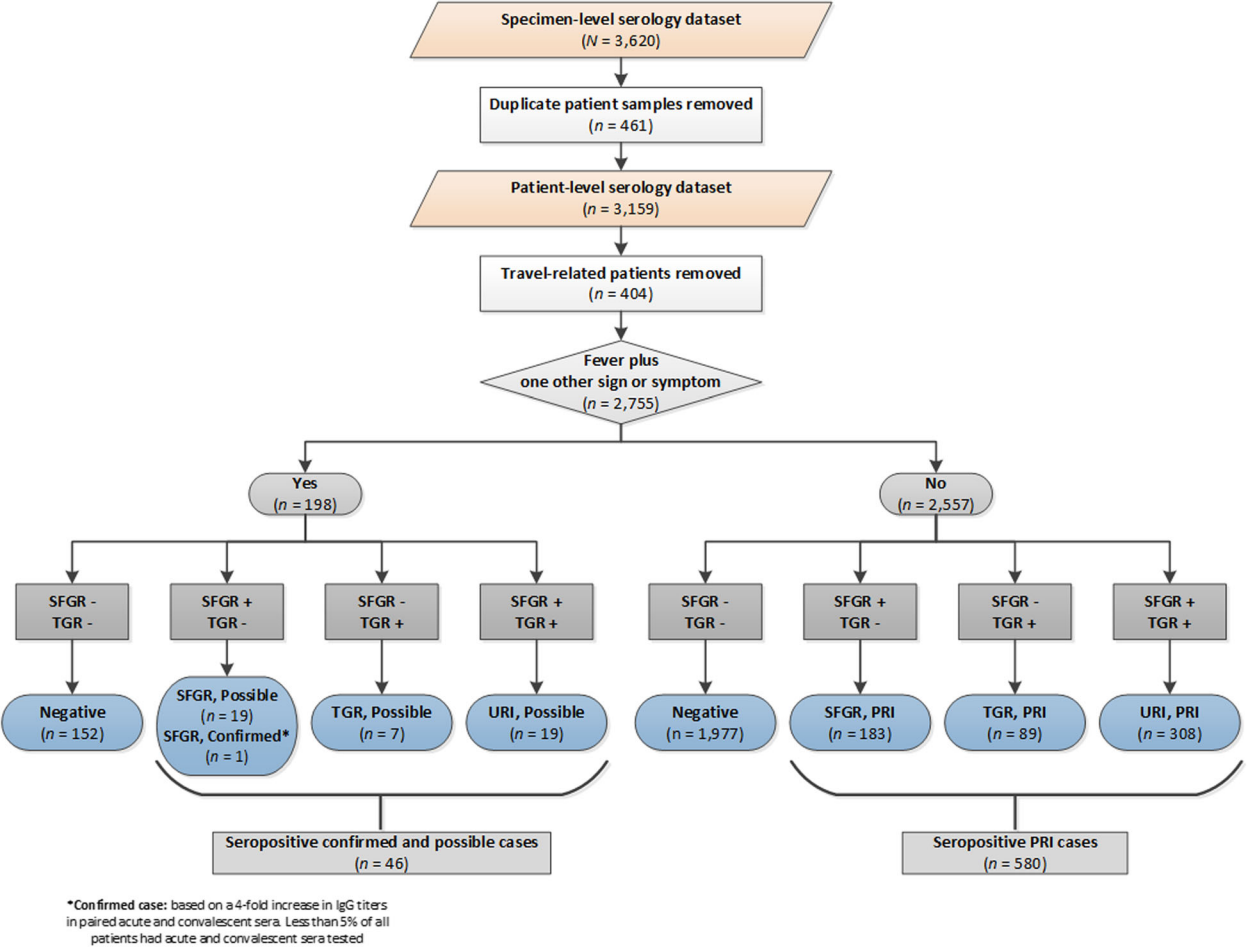
Laboratory diagnoses of SFGR, TGR and URI cases, Ontario, Canada (2013–2018)^a^. *Abbreviations*: +, immunofluorescent assay (IFA) IgG titer ≥1:64 (seropositive); −, IFA IgG titer < 1:64 (seronegative); PRI, previous rickettsial infection; SFGR, spotted fever group rickettsiae; TGR, typhus group rickettsiae; URI, unspecified *Rickettsia* infection. ^a^ We base this decision tree on serology and clinical signs and symptoms. Confirmed cases: 4-fold increase in IgG titers in paired acute and convalescent sera, with evidence of fever plus one other sign or symptom. Possible cases: IgG ≥ 1:64, with evidence of fever and one other sign or symptom. PRI cases: IgG ≥ 1:64, without evidence of fever and one other sign or symptom

From 2013 to 2018, we tested 3620 sera from 3159 patients for rickettsiae (Fig. 1). After excluding 404 patients who reported travel, 2755 patients remained for analysis (see Additional table for information on travel related seropositive and seronegative patients). There was a single confirmed SFGR case, with a 4-fold increase in IFA IgG titers between acute and convalescent sera, and fever, maculopapular rash and headache. There were 19 SFGR, 7 TGR and 19 URI possible cases and 183 SFGR, 89 TGR and 308 URI previous rickettsial infection cases (Tables 2, 3). Overall, 22.7% (626/2755) of all patient sera tested were seropositive (confirmed and possible: 1.7% (46/2755); previous rickettsial infection: 21.1% (580/2755)). The combined seropositive rate (all case classifications for SFGR, TGR and URI) was 4.4 per 100,000 population (confirmed and possible: 0.3/100,000; previous rickettsial infection: 4.1/100,000); for confirmed and possible cases, the seropositive rate for SFGR was 0.14/100,000, TGR (0.050/100,000) and URI (0.14/100,000).

**Table 2:** Summary of confirmed and possible cases of rickettsial infection, Ontario, Canada (2013–2018)

Variable	Spotted fever group rickettsiae^a^	Typhus group rickettsiae	Unspecified *Rickettsia* infection
Total cases, *n*	20	7	19
Female, *n* (%)	13 (65.0)	3 (42.9)	11 (57.9)
Age, mean ± SE (range)	33.3 ± 4.05 (1–62)	45.3 ± 7.66 (19–72)	43.3 ± 5.00 (7–84)
IFA IgG titer, *n*			
1:64	16	5	n/a
1:128	2	1	n/a
1:256	2	1	n/a
IFA SFGR/TGR IgG titers			
1:64/1:64	n/a	n/a	5
1:64/1:128	n/a	n/a	1
1:128/1:64	n/a	n/a	4
1:128/1:128	n/a	n/a	5
1:128/1:256	n/a	n/a	2
1:256/1:128	n/a	n/a	1
1:256/1:256	n/a	n/a	1
Year of onset, *n*			
2013	0	0	1
2014	0	1	1
2015	3	1	2
2016	0	2	6
2017	6	3	4
2018	11	0	5
Signs and symptoms, *n* (%)^b^			
Fever	20 (100.0)	7 (100.0)	19 (100.0)
Headache	10 (50.0)	2 (28.6)	9 (47.4)
Rash (maculopapular)	6 (30.0)	1 (14.3)	2 (10.5)
Gastrointestinal complaints	5 (25.0)	3 (42.9)	6 (31.6)
Rash (vesicular)	4 (20.0)	0 (0.0)	2 (10.5)
Encephalitis/meningitis	4 (20.0)	0 (0.0)	3 (15.8)
Respiratory complaints	3 (15.0)	1 (14.3)	3 (15.8)
Rash (not described)	2 (10.0)	0 (0.0)	2 (10.5)
Tick bite	2 (10.0)	1 (14.3)	0 (0.0)
Fatigue	1 (5.0)	1 (14.3)	7 (36.8)
Malaise/unwell	1 (5.0)	1 (14.3)	3 (15.8)
Arthralgia/arthritis	0 (0.0)	1 (14.3)	2 (10.5)
Chills	0 (0.0)	0 (0.0)	1 (5.3)
Elevated liver enzymes	0 (0.0)	1 (14.3)	0 (0.0)
Myalgia/body aches	0 (0.0)	1 (14.3)	2 (10.5)

*Abbreviations*: *IFA* immunofluorescent assay, *n/a* not applicable, *SFGR* spotted fever group rickettsiae, *TGR* typhus group rickettsiae

^a^ Includes a single confirmed SFGR case

^b^ All cases had evidence of reported fever, as this sign was required for confirmed and possible case classifications. Some cases have > 1 additional sign or symptom

**Table 3:** Summary of previous rickettsial infection cases, Ontario, Canada (2013–2018)

Variable	Spotted fever group rickettsiae	Typhus group rickettsiae	Unspecified *Rickettsia* infection
Total cases, *n*	183	89	308
Female, *n* (%)	93 (50.8)	47 (52.8)	157 (50.9)
Age, mean ± SE (range)	41.3 ± 1.37 (3–87)	44.9 ± 2.11 (2–85)	43.8 ± 1.08 (< 1–89)
IFA IgG titer, *n*			
1:64	154	75	n/a
1:128	22	6	n/a
1:256	7	8	n/a
IFA SFGR/TGR IgG titers (URI)			
1:64/1:64	n/a	n/a	154
1:64/1:128	n/a	n/a	9
1:64/1:256	n/a	n/a	1
1:128/1:64	n/a	n/a	42
1:128/1:128	n/a	n/a	50
1:128/1:256	n/a	n/a	8
1:256/1:64	n/a	n/a	6
1:256/1:128	n/a	n/a	12
1:256/1:256	n/a	n/a	25
1:512/1:512	n/a	n/a	1
Year of onset, *n*			
2013	9	12	19
2014	6	19	22
2015	10	20	33
2016	12	20	36
2017	45	14	92
2018	101	4	106
Signs and symptoms, *n* (%)^a^			
*None reported*	123 (65.1)	65 (73.0)	199 (64.6)
Headache	30 (46.2)	5 (20.8)	53 (39.8)
Fatigue	19 (29.2)	4 (16.7)	33 (24.8)
Arthralgia/arthritis	8 (12.3)	1 (4.2)	7 (5.3)
Fever	7 (10.8)	6 (25.0)	16 (12.0)
Gastrointestinal complaints	6 (9.2)	2 (8.3)	5 (3.8)
Tick bite	4 (6.2)	1 (4.2)	5 (3.8)
Rash (not described)	2 (3.1)	1 (4.2)	6 (4.5)
Respiratory complaints	1 (1.5)	5 (20.8)	8 (6.0)
Elevated liver enzymes	1 (1.5)	0 (0.0)	0 (0.0)
Dizziness	1 (1.5)	0 (0.0)	0 (0.0)
Anemia	1 (1.5)	0 (0.0)	0 (0.0)
Anxiety	1 (1.5)	0 (0.0)	0 (0.0)
Encephalitis/meningitis	1 (1.5)	2 (8.3)	2 (1.5)
Myalgia/body aches	1 (1.5)	0 (0.0)	3 (2.3)
Rash (vesicular)	1 (1.5)	1 (4.2)	2 (1.5)
Rash (maculopapular)	1 (1.5)	3 (12.5)	2 (1.5)
Jaundice	1 (1.5)	0 (0.0)	1 (0.8)
Malaise/unwell	0 (0.0)	0 (0.0)	4 (3.0)
Chills	0 (0.0)	0 (0.0)	1 (0.8)

*Abbreviations*: *IFA* immunofluorescent assay, *n/a* not applicable, *SFGR* spotted fever group rickettsiae, *TGR* typhus group rickettsiae

^a^ Cases reporting at least one sign or symptom: SFGR, *n* = 60; TGR, *n* = 24; URI, *n* = 109. Some cases have > 1 sign or symptom. Denominator is based on number of cases with at least one sign or symptom reported on the requisition

Less than 5% (136/2755) of all patients had acute and convalescent sera tested. A higher proportion of seropositive patients (positive serology on acute and/or convalescent sera: 10.2%, 64/626) had paired acute and convalescent sera tested than seronegative patients (3.4%, 72/2129) (chi-square test: *χ*^2^ = 48.3, *df* = 1, *p* < 0.00001). The percent of seropositive (38.2%) and seronegative (36.1%) patients reporting at least one sign or symptom was low; therefore, it was not appropriate to test for between-group differences in specific signs and symptoms. In addition to fever (which was required to meet the definition for confirmed and possible cases), other symptoms reported by confirmed and possible cases included headache (45.7%) gastrointestinal complaints (30.4%) and maculopapular rash (19.6%).

### Annual and seasonal trends

**Fig. 2:**
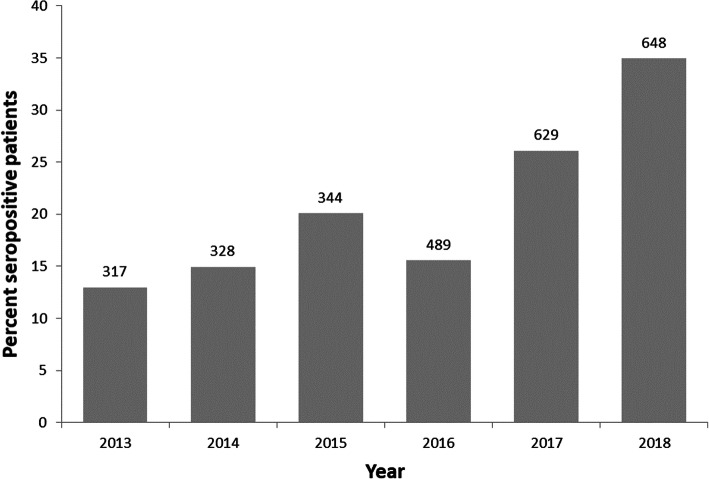
Annual percent of patients tested who are seropositive for rickettsiae (all case classifications for SFGR, TGR an URI combined) (total patients tested each year indicated above each bar), Ontario, Canada (2013–2018)

The percent of patients per year (all case classifications for SFGR, TGR, URI combined) that were seropositive increased during the study, as did the number of tests for rickettsiae performed per year (Fig. 2). In addition, odds of having new cases (all case classifications for SFGR, TGR, URI combined) increased by 30% annually (odds ratio for total number of cases (OR) = 1.3, 95% confidence interval (CI): 1.23–1.39); an OR of 1.3 means the odds of having new cases increased by 30% annually. The annual number of cases (all case classifications combined) increased for SFGR (OR = 1.5, 95% CI: 1.39–1.60), TGR (OR = 1.1, 95% CI: 1.05–1.19) and URI (OR = 1.3, 95% CI: 1.18–1.38).

**Fig. 3:**
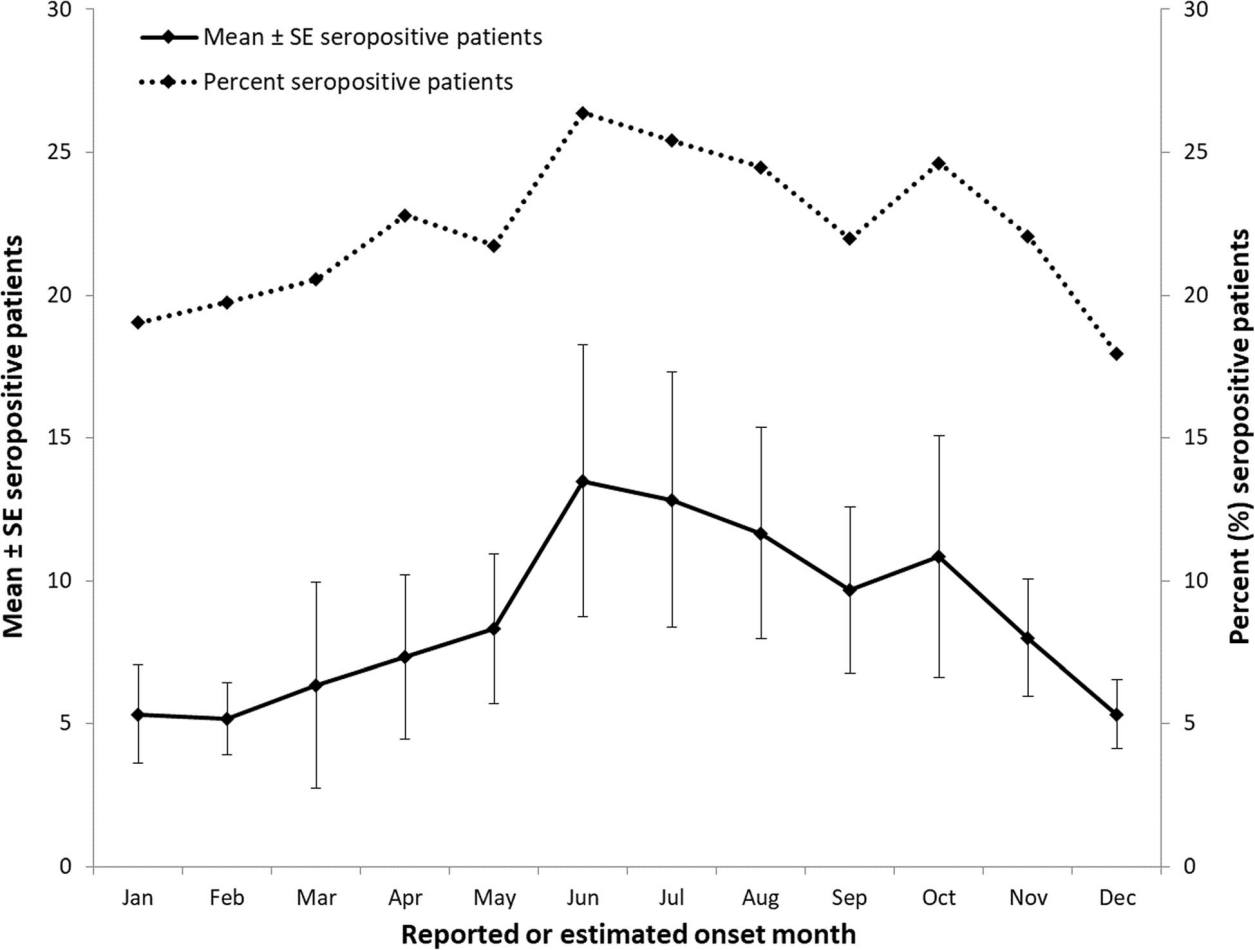
Reported or estimated symptom onset month for rickettsiae cases (all case classifications for SFGR, TGR and URI combined), Ontario, Canada (2013–2018)

Approximately 6.1% of seropositive patients reported a symptom onset date and for the remainder the onset date was estimated based on the specimen collection date. Most cases had a reported or estimated onset date in summer (June–August: 36.4%, 228/626) and fall (September–November: 27.2%, 170/626) (Fig. 3). Using likelihood ratio test, the monthly number of cases (all case classifications for SFGR, TGR, URI combined) did not show a statistically significant variation by season (*p* = 0.16) (SFGR: *p* = 0.70, TGR: *p* = 0.42, URI: *p* = 0.22).

### Sex and age

The percent of seronegative patients who were female (58.8%, 1253/2129) was higher than percent of seropositive cases who were female (47.4%, 297/626) (*χ*^2^ = 31.5, *df* = 1, *p* < 0.0001) (Table 4). There was no statistically significant difference in the percentage of cases by sex among confirmed and possible SFGR, TGR and URI cases (*χ*^2^ = 1.1, *df* = 4, *p* = 0.90) or previous rickettsial infection SFGR, TGR and URI cases (*χ*^2^ = 1.8, *df* = 4, *p* = 0.77) (Tables 2, 3). The mean age of all seropositive cases (42.9 ± 0.76 years) was similar to seronegative patients (43.0 ± 0.40 years) (ANOVA: *F*_(1,2740)_ = 0.007, *p* = 0.94) (Table 4). There was no difference in mean age among confirmed and possible SFGR, TGR and URI cases (*F*_(2,43)_ = 1.6, *p* = 0.21) or previous rickettsial infection SFGR, TGR and URI cases (*F*_(2,575)_ = 1.4, *p* = 0.25) (Tables 2, 3).

**Table 4:** Summary of rickettsiae-seropositive and seronegative cases and patients, Ontario, Canada (2013–2018)

Variable	Seropositive cases	Seronegative patients
Total, *n*	626	2129
Female, *n* (%)	297 (47.4)	1253 (58.8)
Age, mean ± SE (range)^a^	42.9 ± 0.76 (< 1–89)	43.0 ± 0.40 (< 1–90)
Sera samples/patient, mean ± SE (range)^b^	1.3 ± 0.026 (1–7)	1.1 ± 0.0067 (1–4)
Year of onset, *n*		
2013	41	276
2014	49	279
2015	69	275
2016	76	413
2017	164	465
2018	227	421
Signs and symptoms, *n* (%)^c^		
*None reported*	387 (61.8)	1361 (63.9)
Headache	134 (54.0)	263 (33.1)
Fever	75 (30.2)	253 (31.8)
Fatigue	65 (26.2)	225 (28.3)
Gastrointestinal complaints	27 (10.9)	86 (10.8)
Respiratory complaints	21 (8.5)	59 (7.4)
Arthralgia/arthritis	19 (7.7)	65 (8.2)
Rash (maculopapular)	15 (6.0)	79 (9.9)
Rash (not described)	13 (5.2)	51 (6.4)
Tick bite	13 (5.2)	46 (5.8)
Encephalitis/meningitis	12 (4.8)	23 (2.9)
Rash (vesicular)	10 (4.0)	27 (3.4)
Malaise/unwell	9 (3.6)	5 (0.6)
Myalgia/body aches	7 (2.8)	10 (1.3)
Chills	2 (0.8)	8 (1.0)
Elevated liver enzymes	2 (0.8)	7 (0.9)
Jaundice	2 (0.8)	5 (0.6)
Anemia	1 (0.4)	2 (0.3)
Anxiety	1 (0.4)	2 (0.3)
Dizziness	1 (0.4)	5 (0.6)
Confusion	0 (0.0)	2 (0.3)
Hepatitis	0 (0.0)	2 (0.3)
Edema	0 (0.0)	1 (0.1)

*Abbreviations*: *n/a* not applicable

^a^ Sex of some patients was not available

^b^*t* = 7.3, *df* = 706, *p* < 0.0001

^c^ Cases and patients reporting at least one sign or symptom: seropositive, *n* = 239; seronegative, *n* = 768. Some cases and patients have > 1 sign or symptom. Denominator is based on number of cases or patients with at least one symptom reported on the requisition

### Geographical distribution

**Fig. 4:**
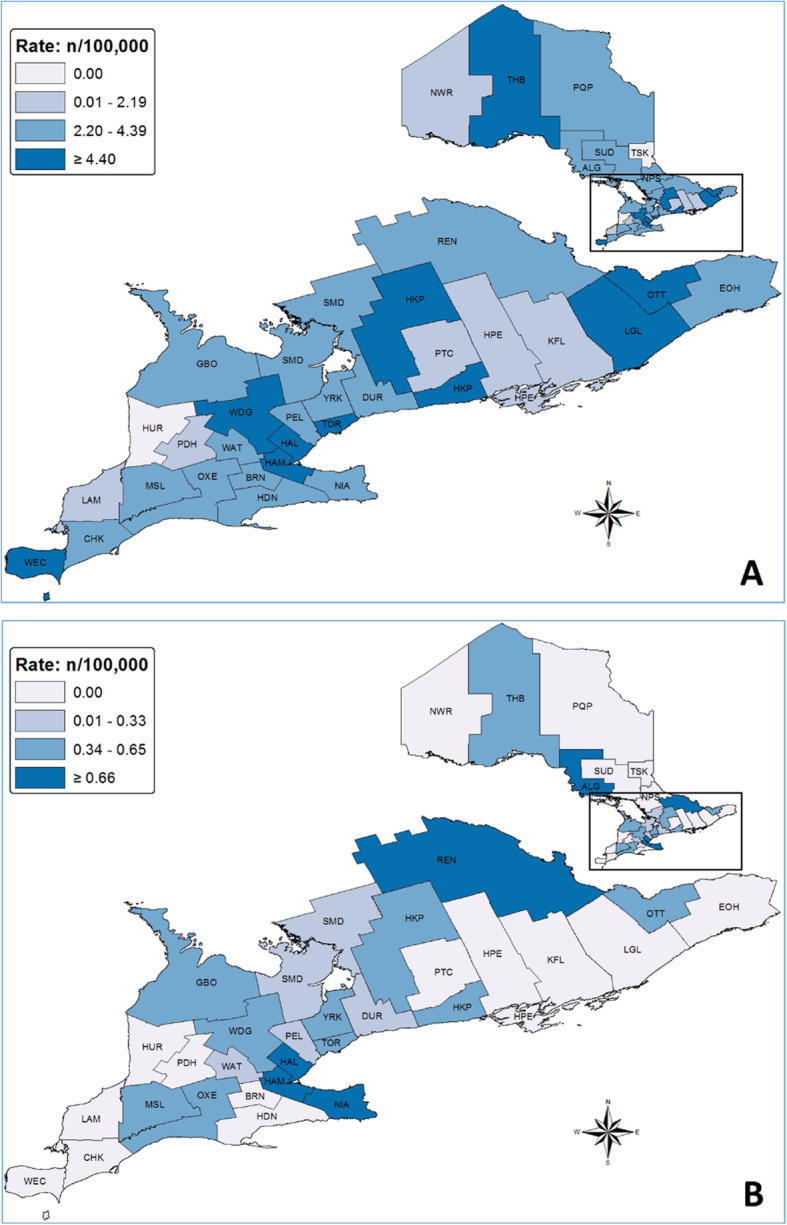
SFGR, TGR and URI rickettsiae case rates for (**a**) all case classifications combined and (**b**) confirmed and possible cases only, Ontario, Canada (2013–2018). The maps are our own and created by Public Health Ontario. *Abbreviations*: ALG, Algoma District; BRN, Brant County; CHK, Chatham-Kent; DUR, Durham Regional; EOH, Eastern Ontario; GBO, Grey Bruce; HAL, Halton Regional; HAM, City of Hamilton; HDN, Haldimand-Norfolk; HKP, Haliburton-Kawartha-Pine Ridge District; HPE, Hastings and Prince Edward Counties; HUR, Huron County; KFL, Kingston-Frontenac and Lennox & Addington; LAM, Lambton; LGL, Leeds-Grenville and Lanark District; MSL, Middlesex-London; NIA, Niagara Regional; NPS, North Bay Parry Sound District; NWR, Northwestern; OTT, City of Ottawa; OXE, Oxford Elgin-St. Thomas; PDH, Perth District; PEL, Peel Regional; PQP, Porcupine; PTC, Peterborough County-City; REN, Renfrew County and District; SFGR, spotted fever group rickettsiae; SMD, Simcoe Muskoka District; SUD, Sudbury and District; TGR, typhus group rickettsiae; THB, Thunder Bay District; TOR, City of Toronto; TSK, Timiskaming; URI, unspecified *Rickettsia* infection; WAT, Waterloo; WDG, Wellington-Dufferin-Guelph; WEC, Windsor-Essex County; YRK, York Regional

The rate for all cases combined (all case classifications for SFGR, TGR, URI) was highest in HAL, HAM, HKP, LGL, OTT, THB, TOR, WDG and WEC (≥ 4.4/100,000) (Fig. 4). For confirmed and possible cases (SFGR, TGR and URI combined), rates were highest in ALG, HAL, HAM, NIA and REN (≥ 0.66/100,000). Using likelihood ratio tests, the total number of cases by PHU (all case classifications for SFGR, TGR, URI combined) did not show a statistically significant variation by PHU (*p* = 0.64) (SFGR: *p* = 0.76, TGR: *p* = 0.51, URI: *p* = 0.68).

## Discussion

We assessed the serological status of SFGR and TGR in 2755 non-travel patients in Ontario from 2013 through 2018. We detected one confirmed SFGR case, who demonstrated a 4-fold increase in IgG titers between acute and convalescent sera, with evidence of fever, maculopapular rash and headache. There were 45 possible cases (19 SFGR, 7 TGR, 19 URI) and 580 previous rickettsial infection cases (183 SFGR, 89 TGR, 308 URI). The rate for all cases combined was 4.4 per 100,000 population (confirmed and possible: 0.3/100,000; previous rickettsial infection: 4.1/100,000). We expected a low number of confirmed infections, given less than 5% of patients had the appropriately paired acute and convalescent sera tested, that are required for confirming a case. In the USA (2010–2015), 1% of 16,807 SFGR cases were confirmed based on evidence of increased IgG titers in paired sera, whereas, 99% of cases were probable based on a single elevated IgG titer [35]. Testing of paired sera is important for estimating SFGR and TGR incidence in Ontario, especially since the province is a non-endemic region where the underlying seroprevalence in the population is unknown. We suspect most patients were treated empirically based on the initial elevated titre and clinical presentation and testing of convalescent sera was not requested. It is difficult to determine the rate of rickettsial disease in Ontario. The numbers of confirmed and possible infections would suggest it is low; however, the number of previous rickettsial infection cases could indicate a higher rate of infection if symptoms compatible with the possible case definition were present but not appropriately documented on the requisition form.

The highest rates of confirmed and possible (SFGR, TGR and URI combined) cases were from diverse landscapes and populations, including from PHUs considered primarily rural (Algoma District, Renfrew County and District), to rural/urban (Niagara Regional), suburban (Halton Regional) and urban (City of Hamilton). These PHUs with high rates of confirmed and possible cases also have high or increasing submission rates of *D. variabilis* (based on Ontario’s passive tick surveillance program), a potential vector of SFGR in the province (MPN, unpublished data). In 2017, the rate of laboratory-diagnosed confirmed and possible SFGR cases in Ontario (0.14 per 100,000) was similar to the relatively low SFGR case rates in neighbouring New York (0.19/100,000) and Michigan (0.13/100,000) from the same year [36, 37]. Future work in Ontario could include spatial and temporal correlations between human rickettsial rates by PHU and the number of submitted American dog ticks and their percent positivity for rickettsial pathogens.

*Rickettsia* IFA IgG serology is group, rather than species, specific; therefore, we can only speculate on *Rickettsia* species responsible for seropositivity in Ontario patients. In 1979, there was a non-travel RMSF case reported near Ottawa, the only case ever reported in the province [12]. In 2006, a dog from Ottawa, with no travel history, was seropositive for *R*. *rickettsii* [13]. *Rickettsia rickettsii* detections in Ontario ticks have been from American dog ticks and rabbit ticks (*Haemaphysalis leporispalustris*), all prior to 1973 [14, 15]. A 2016 study of Ontario American dog ticks did not detect *R*. *rickettsii*, but did detect *R*. *montanensis* [16]. Based on these studies, there is no evidence supporting the presence of *R*. *rickettsii* in Ontario; however, other *Rickettsia* species, such as *R. montanensis*, could be responsible for seroreactivity in patients. *R*. *montanensis*, initially considered non-pathogenic, is a potential spotted fever agent in the southern USA [21]. Recently, *Rickettsia akari* (rickettsialpox) infection was reported in Ontario for the first time, but studies of vector mites and reservoirs are lacking in the province [17]. Researchers have detected *R*. *felis* from cat fleas (ex domestic cats) in Ontario; however, no human infections have been reported [18]. Flea-borne spotted fever, caused by *R*. *felis*, is a relatively mild disease and infections likely go undetected because patients do not seek out medical attention [38]. Rickettsial agents responsible for seroreactive patients in Ontario remain unknown and further studies identifying specific rickettsial agents in the province’s vectors and clinical samples is warranted.

We expected the low prevalence of TGR-seropositive patients, as we know of no reports of *R*. *prowazekii* or *R*. *typhi* in Ontario vectors, reservoirs or humans. Murine typhus is infrequent in North America, but recent outbreaks in southern Texas and California involved cats, Virginia opossums and their fleas [39, 40]. The only epidemic typhus outbreak in Canada occurred in 1847 (New Brunswick, Ontario, Québec), during the Great Famine (1845–1849) and the mass immigration of Irish to Canada [41]. Recently, research using insurance claims as a data source suggests the incidence of TGR infections is higher in throughout the USA than previously thought [42]. Assessing the mites, lice and fleas of Ontario rodents for rickettsiae would add additional information on the potential for human infection, as we are not aware of any such studies in the province.

We note several limitations in our research, besides those already mentioned; however, these limitations represent opportunities for further research on rickettsiae in Ontario (vectors and reservoirs). *Rickettsia* IFA IgG serology is the gold standard for laboratory diagnosis of rickettsial infections, with a sensitivity > 90% and a specificity of 100% after 14 days since symptom onset [43]. Nonetheless, in a low prevalence setting, such as Ontario, we would expect some positives to be false positives, resulting in a lower positive predictive value of serology testing than in higher prevalence settings. As with other tick-borne serology tests, rickettsial serology is often negative in early infection; therefore, we would expect to miss infections in Ontario patients tested in the first week of illness. In contrast, we have likely overestimated rickettsial exposure, since we used sensitive case definitions for possible and previous rickettsial infection cases relying on single elevated IgG titers (≥ 1:64), with or with clinical evidence of infection respectively [32]. It was difficult to document 4-fold increases in IgG titers in our study since our endpoint titer was relatively low at 1:256, meaning we likely underestimated confirmed cases. A higher endpoint titre may have been able to demonstrate more seroconversions among those who had acute and convalescent sera tested. Given the relatively high number of cases classified as previous rickettsial infection, there is a possibility that the rickettsial disease rate is underestimated if symptoms are not accurately captured on the laboratory requisition. In addition, the previous rickettsial infection cases highlight the need for better adherence to including clinical information on laboratory requisitions for serological testing. On laboratory requisitions, travel information is not well-documented and was restricted to recent travel only; therefore, positive serology could be a result of past exposure as IFA IgG titers can remain elevated for over 12 months post-exposure [3]. Estimated exposure dates occur mostly during the summer months and align with potential vector activity; however, readers should use caution when interpreting these results due to the combination of low numbers of cases with symptom onset dates (< 5%) and the potential persistence of IgG from earlier infections. We suggest further epidemiological studies of *Rickettsia* infections in Ontario, especially with the use of more specific testing methods, such as using species-specific serology or PCR on blood or tissue samples (e.g., skin biopsy or eschar swab).

## Conclusions

The rate of rickettsial infection in humans in Ontario is difficult to determine. Based on confirmed and possible definitions, the rate would appear to be low, but could be higher due to previous rickettsial infection cases. We emphasize the importance of testing acute and convalescent sera in confirming rickettsial disease in Ontario and consistently reporting clinical symptoms on the requisition. We recommend further field and clinical research into potential rickettsial agents in Ontario, especially in areas where there are higher rates of human infection as determined by this study.

## Supplementary information

**Additional file 1: Additional Table.** Summary of travel-related seropositive and seronegative patients

## Data Availability

Information about PHO’s data request process is available on-line at https://www.publichealthontario.ca/en/data-and-analysis/using-data/data-requests

## Abbreviations

AGR: Ancestral group rickettsiae
ALG: Algoma District
BRN: Brant County
CHK: Chatham-Kent
CI: Confidence interval
DUR: Durham Regional
EOH: Eastern Ontario
GBO: Grey Bruce
HAL: Halton Regional
HAM: City of Hamilton
HDN: Haldimand-Norfolk
HKP: Haliburton-Kawartha-Pine Ridge District
HPE: Hastings and Prince Edward Counties
HUR: Huron County
IFA: Immunofluorescence assay
KFL: Kingston-Frontenac and Lennox & Addington
LAM: Lambton
LGL: Leeds-Grenville and Lanark District
MSL: Middlesex-London
NIA: Niagara Regional
NPS: North Bay Parry Sound District
NWR: Northwestern
OR: Odds ratio
OTT: City of Ottawa
OXE: Oxford Elgin-St. Thomas
PDH: Perth District
PHU: Public health unit
PEL: Peel Regional
PQP: Porcupine
PTC: Peterborough County-City
REN: Renfrew County and District
RMSF: Rocky Mountain spotted fever
SMD: Simcoe Muskoka District
SFGR: Spotted fever group rickettsiae
SUD: Sudbury and District
TGR: Typhus group rickettsiae
THB: Thunder Bay District
TOR: City of Toronto
TSK: Timiskaming
WAT: Waterloo
WDG: Wellington-Dufferin-Guelph
WEC: Windsor-Essex County
YRK: York Regional
