# Supplementary material for: Assessing human exposure to spotted fever and typhus group rickettsiae in Ontario, Canada (2013–2018): a retrospective, cross-sectional study
Source: BMC Infect Dis. 2020 Jul 18;20:523. doi: 10.1186/s12879-020-05244-8 (PMC7368706; doi:10.1186/s12879-020-05244-8)
Supplement: Supplementary file 1 — Additional file 1: Additional Table. Summary of travel-related seropositive and seronegative patients [file 12879_2020_5244_MOESM1_ESM.docx]

**Additional Table** Summary of travel-related seropositive cases and seronegative patients based on rickettsiae testing, Ontario, Canada (2013–2018)

| Variable | Seropositive cases^a^ | | | Seronegative patients |
| --- | --- | --- | --- | --- |
|  | **SFGR** | **TGR** | **URI** |  |
| Total, *n* | 36 | 5 | 68 | 295 |
| Female, *n* (%) | 20 (55.6) | 2 (40.0) | 36 (52.9) | 164 (55.6) |
| Age, mean ± SE | 42.5 ± 2.86 | 28.0 ± 7.31 | 42.0 ± 2.10 | 44.1 ± 1.00 |
| IFA IgG titer, *n* |  |  |  |  |
| 1:64 | 28 | 5 | n/a | n/a |
| 1:128 | 6 | 0 | n/a | n/a |
| 1:256 | 2 | 0 | n/a | n/a |
| IFA SFGR/TGR IgG titers for URIs, *n* | | | | |
| 1:64/1:64 | n/a | n/a | 26 | n/a |
| 1:64/1:256 | n/a | n/a | 1 | n/a |
| 1:128/1:64 | n/a | n/a | 8 | n/a |
| 1:128/1:128 | n/a | n/a | 15 | n/a |
| 1:256/1:128 | n/a | n/a | 8 | n/a |
| 1:256/1:256 | n/a | n/a | 7 | n/a |
| Year of onset or year tested, *n* | | | | |
| 2013 | 6 | 0 | 1 | 34 |
| 2014 | 2 | 1 | 6 | 30 |
| 2015 | 0 | 3 | 3 | 32 |
| 2016 | 1 | 1 | 6 | 57 |
| 2017 | 8 | 0 | 22 | 52 |
| 2018 | 19 | 0 | 30 | 89 |
| Onset month (reported or estimated), *n* | | | | |
| January | 0 | 0 | 2 | 8 |
| February | 2 | 0 | 4 | 20 |
| March | 6 | 0 | 2 | 24 |
| April | 2 | 1 | 8 | 32 |
| May | 3 | 0 | 8 | 30 |
| June | 3 | 2 | 4 | 36 |
| July | 4 | 0 | 8 | 26 |
| August | 2 | 2 | 7 | 28 |
| September | 6 | 0 | 6 | 20 |
| October | 6 | 0 | 6 | 26 |
| November | 0 | 0 | 1 | 22 |
| December | 2 | 0 | 7 | 23 |
| Signs and symptoms, *n*^b^ | | | | |
| *None reported* | 9 (25.0) | 1 (20.0) | 18 (26.5) | 59 (20.0) |
| Fever | 17 (63.0) | 2 (50.0) | 18 (36.0) | 118 (50.0) |
| Headache | 10 (37.0) | 1 (25.0) | 21 (42.0) | 81 (34.3) |
| Fatigue | 5 (18.5) | 0 (0.0) | 19 (38.0) | 74 (31.4) |
| Gastrointestinal complaints | 5 (18.5) | 0 (0.0) | 5 (10.0) | 41 (17.4) |
| Arthralgia/arthritis | 4 (14.8) | 0 (0.0) | 4 (8.0) | 26 (11.0) |
| Rash (maculopapular) | 6 (22.2) | 1 (25.0) | 2 (4.0) | 32 (13.6) |
| Respiratory complaints | 3 (11.1) | 1 (25.0) | 4 (8.0) | 18 (7.6) |
| Rash (vesicular) | 4 (14.8) | 0 (0.0) | 3 (6.0) | 14 (5.9) |
| Encephalitis/meningitis | 1 (3.7) | 0 (0.0) | 4 (8.0) | 16 (6.8) |
| Tick bite | 1 (3.7) | 0 (0.0) | 3 (6.0) | 11 (4.7) |
| Liver function abnormalities | 1 (3.7) | 0 (0.0) | 0 (0.0) | 1 (0.4) |
| Myalgia | 0 (0.0) | 0 (0.0) | 1 (2.0) | 5 (2.1) |
| Rash (not described) | 0 (0.0) | 0 (0.0) | 1 (2.0) | 16 (16.8) |
| Chills | 0 (0.0) | 0 (0.0) | 1 92.0) | 2 (0.8) |

*Abbreviations*: IFA, immunofluorescent assay; n/a, not applicable; SFGR, spotted fever group rickettsiae; TGR, typhus group rickettsiae; URI, unspecified *Rickettsia* infection

^a^ Reported location of travel. SFGR: Africa = 11; Europe = 8; Central and South America = 7; Asia = 5; USA = 4; unknown = 4; elsewhere in Canada = 1. TGR: Asia = 2; Central and South America = 2; unknown = 1. URI: Europe = 26; Caribbean, Central and South America = 11; Africa = 9; Asia = 9; unknown = 9; USA = 7; elsewhere in Canada = 1.

^b^ Cases and patients reporting at least one sign or symptom: SFGR, *n* = 27; TGR, *n* = 4; URI, *n* = 50; seronegative patients, *n* = 236. Some aces and patients have > 1 sign or symptom. Denominator is based on number of cases or patients with at least one symptom reported on the requisition.
